# Exposure to outdoor air pollution and its human health outcomes: A scoping review

**DOI:** 10.1371/journal.pone.0216550

**Published:** 2019-05-16

**Authors:** Zhuanlan Sun, Demi Zhu

**Affiliations:** 1 Department of Management Science and Engineering, School of Economics and Management, Tongji University, Shanghai, China; 2 Department of Comparative Politics, School of International and Public Affairs, Shanghai Jiaotong University, Shanghai, China; University of Lille, FRANCE

## Abstract

Despite considerable air pollution prevention and control measures that have been put into practice in recent years, outdoor air pollution remains one of the most important risk factors for health outcomes. To identify the potential research gaps, we conducted a scoping review focused on health outcomes affected by outdoor air pollution across the broad research area. Of the 5759 potentially relevant studies, 799 were included in the final analysis. The included studies showed an increasing publication trend from 1992 to 2008, and most of the studies were conducted in Asia, Europe, and North America. Among the eight categorized health outcomes, asthma (category: respiratory diseases) and mortality (category: health records) were the most common ones. Adverse health outcomes involving respiratory diseases among children accounted for the largest group. Out of the total included studies, 95.2% reported at least one statistically positive result, and only 0.4% showed ambiguous results. Based on our study, we suggest that the time frame of the included studies, their disease definitions, and the measurement of personal exposure to outdoor air pollution should be taken into consideration in any future research. The main limitation of this study is its potential language bias, since only English publications were included. In conclusion, this scoping review provides researchers and policy decision makers with evidence taken from multiple disciplines to show the increasing prevalence of outdoor air pollution and its adverse effects on health outcomes.

## Introduction

In recent years, despite considerable improvements in air pollution prevention and control, outdoor air pollution has remained a major environmental health hazard to human beings. In some developing countries, the concentrations of air quality far exceed the upper limit announced in the World Health Organization guidelines [1]. Moreover, it is widely acknowledged that outdoor air pollution increases the incidence rates of multiple diseases, such as cardiovascular disease, lung cancer, respiratory symptoms, asthma, negatively affected pregnancy, and poor birth outcomes [2–6].

The influence of outdoor air pollution exposure and its mechanisms continue to be hotly debated [7–11]. Some causal inference studies have been conducted to examine these situations [12]; these have indicated that an increase in outdoor air exposure affects people’s health outcomes both directly and indirectly [13]. However, few studies in the existing literature have examined the extent, range, and nature of the influence of outdoor air pollution with regard to human health outcomes. Thus, such research gaps need to be identified, and related fields of study need to be mapped.

Systematic reviews and meta-analyses, the most commonly used traditional approach to synthesize knowledge, use quantified data from relevant published studies in order to aggregate findings on a specific topic [14]; furthermore, they formally assesses the quality of these studies to generate precise conclusions related to the focused research question [15]. In comparison, scoping review is a more narrative type of knowledge synthesis, and it focuses on a broader area [16] of the evidence pertaining to a given topic. It is often used to systematically summarize the evidence available (main sources, types, and research characteristics), and it tends to be more comprehensive and helpful to policymakers at all levels.

Scoping reviews have already been used to examine a variety of health related issues [17]. As an evidence synthesis approach that is still in the midst of development, the methodology framework for scoping reviews faces some controversy with regard to its conceptual clarification and definition [18,19], the necessity of quality assessment [20–22], and the time required for completion [19,21,23]. Comparing this approach with other knowledge synthesis methods, such as evidence gap map and rapid review, the scoping review has become increasingly influential for efficient evidence-based decision-making because it offers a very broad topic scope [15].

To our knowledge, few studies have systematically reviewed the literature in the broad field of outdoor air pollution exposure research, especially with regard to related health outcomes. To fill this gap, we conducted a comprehensive scoping review of the literature with a focus on health outcomes affected by outdoor air pollution. The purposes of this study were as follows: 1) provide a systematic overview of relevant studies; 2) identify the different types of outdoor air pollution and related health outcomes; and 3) summarize the publication characteristics and explore related research gaps.

## Materials and methods

The methodology framework used in this study was initially outlined by Arksey and O’Malley [23] and further advanced by Levac et al. [20], Daudt et al. [21], and the Joanna Briggs Institute [24]. The framework was divided into six stages: identifying the research question; identifying relevant studies; study selection; charting the data; collating, summarizing and reporting the results; and consulting exercise.

### Stage one: Research question identification

As recommended, we combined broader research questions with a clearly articulated scope of inquiry [20]; this included defining the concept, target population, and outcomes of interest in order to disseminate an effective search strategy. Thus, an adaptation of the “PCC” (participants, concept, context) strategy was used to guide the construction of research questions and inclusion criteria [24].

#### Types of participants

There were no strict restrictions on ages, genders, ethnicity, or regions of participants. Everyone, including newborns, children, adults, pregnant women, and the elderly, suffer from health outcomes related to exposure to outdoor air pollution; hence, all groups were included in the study to ensure that the inquiry was sufficiently comprehensive.

#### Concept

The core concept was clearly articulated in order to guide the scope and breadth of the inquiry [24]. A list of outdoor air pollution and health outcome related terms were compiled by reviewing potential text words in the titles or abstracts of the most pertinent articles [25–33]; we also read the most cited literature reviews on air pollution related health outcomes. To classify the types of air pollution and health outcomes, we consulted researchers from different air pollution related disciplines. The classified results are shown in Table 1.

**Table 1:** Outdoor air pollutants and aspects of health outcomes.

Concept	Category	Detailed Substance
**Outdoor air pollution**	General air pollution gas	Ozone (O_3_), sulfur dioxide (SO_2_), carbon monoxide (CO), nitrogen dioxide (NO_2_)
	Fine particulate matter	Total suspended particle, suspended particulate matter, PM_2.5_, PM_10_
	Other hazardous substances	Toxic air pollutants, volatile organic pollutants, nitrogen oxides (NO_x_)
**Health outcomes**	Respiratory diseases	Asthma, lung cancer, respiratory infections, respiratory disorder, chronic obstructive pulmonary disease
	Chronic diseases	diabetes, chronic respiratory diseases
	Cardiovascular diseases	Hypertension, heart rate variability, heart attack, cardiopulmonary disease, ischemic heart disease, blood coagulation, deep vein thrombosis, stroke
	Health records	Morbidity, hospital admissions, outpatient visits, emergency room visits, mortality
	Other diseases	DNA methylation changes, neurobehavioral functions, inflammatory disease, skin disease, abortion, Alzheimer’s disease, disability, cognitive function, Parkinson’s disease

#### Context

Our scoping review included studies from peer-reviewed journals. There were no restrictions in terms of the research field, time period, and geographical coverage. The intended audiences of our scoping review were researchers, physicians, and public policymakers.

### Stage two: Relevant studies identification

We followed Joanna Briggs Institute’s instructions [24] to launch three-step search strategies to identify all relevant published and unpublished studies (grey literature) across the multi-disciplinary topic in an iterative way. The first step included a limited search of the entire database using keywords relevant to the topic and conducting an abstract and indexing categorizations analysis. The second step was a further search of all included databases based on the newly identified keywords and index terms. The final step was to search the reference list of the identified reports and literatures.

#### Electronic databases

We conducted comprehensive literature searches by consulting with an information specialist. We searched the following three electronic databases from their inception until now: PubMed, Web of Science, and Scopus. The language of the studies included in our sample was restricted to English.

#### Search terms

The search terms we used were broad enough to uncover any related literature and prevent chances of relevant information being overlooked. This process was conducted iteratively with different search item combinations to ensure that all relevant literature was captured (S1 Table).

The search used combinations of the following terms: 1) outdoor air pollution (ozone, sulfur dioxide, carbon monoxide, nitrogen dioxide, PM_2.5_, PM_10_, total suspended particle, suspended particulate matter, toxic air pollutant, volatile organic pollutant, nitrogen oxide) and 2) health outcomes (asthma, lung cancer, respiratory infection, respiratory disorder, diabetes, chronic respiratory disease, chronic obstructive pulmonary disease, hypertension, heart rate variability, heart attack, cardiopulmonary disease, ischemic heart disease, blood coagulation, deep vein thrombosis, stroke, morbidity, hospital admission, outpatient visit, emergency room visit, mortality, DNA methylation change, neurobehavioral function, inflammatory disease, skin disease, abortion, Alzheimer’s disease, disability, cognitive function, Parkinson’s disease).

#### Additional studies search

Key, important, and top journals were read manually, reference lists and citation tracing were used to collect studies and related materials, and suggestions from specialists were considered to guarantee that the research was as comprehensive as possible.

Bibliographies Management Software (Mendeley) was used to remove duplicated literatures and manage thousands of bibliographic references that needed to be appraised to check whether they should be included in the final study selection.

Our literature retrieval generated a total of 5759 references; the majority of these (3567) were found on the Scopus electronic database, which emphasized the importance of collecting the findings on this broad topic.

### Stage three: Studies selection

Our study identification picked up a large number of irrelevant studies; we needed a mechanism to include only the studies that best fit the research question. The study selection stage should be an iterative process of searching the literature, refining the search strategy, and reviewing articles for inclusion. Study inclusion and exclusion criteria were discussed by the team members at the beginning of the process, then two inter-professional researchers applied the criteria to independently review the titles and abstracts of all studies [21]. If there were any ambiguities, the full article was read to make decision about whether it should be chosen for inclusion. When disagreements on study inclusion occurred, a third specialist reviewer made the final decision. This process should be iterative to guarantee the inclusion of all relevant studies.

#### Inclusion and exclusion criteria

The inclusion criteria used in our scoping study ensured that the articles were considered only if they were: 1) long-term and short-term exposure, perspective or prospective studies; 2) epidemiological time series studies; 3) meta-analysis and systematic review articles rather than the primary studies that contained the main parameters we were concerned with; 4) economic research studies using causality inference with observational data; and 5) etiology research studies on respiratory disease, cancer, and cardiovascular disease.

Articles were removed if they 1) focused exclusively on indoor air pollution exposure and 2) did not belong to peer-reviewed journals or conference papers (such as policy documents, proposals, and editorials).

### Stage four: Data charting

The data extracted from the final articles were entered into a “data charting form” using the database, programmed Excel, so that the following relevant data could be recorded and charted according to the variables of interest (Table 2).

**Table 2:** Key information to be charted during the review process.

Study Characteristics	Air Pollution Characteristics	Participant Characteristics	Key Findings
Author (s)	Type of air pollution	Study region	Primary outcome
Year of publication		Target population	
Type of publication		Total sample size	
Discipline		Type of disease	
Continent^a^			
Study location			
Study method			

^a^ The classification of continent was based on the address of the corresponding author.

### Stage five: Results collection, summarization, and report

The extracted data were categorized into topics such as people’s health types and regions of diseases caused by outdoor air exposure. Each reported topic should be provided with a clear explanation to enable future research. Finally, the scoping review results were tabulated in order to find research gaps to either enable meaningful research or obtain good pointers for policymaking.

### Stage six: Consultation exercise

Our scoping review took into account the consultation phase of sharing preliminary findings with experts, all of whom are members of the Committee on Public Health and Urban Environment Management in China. This enabled us to identify additional emerging issues related to health outcomes.

## Results

**Fig 1:**
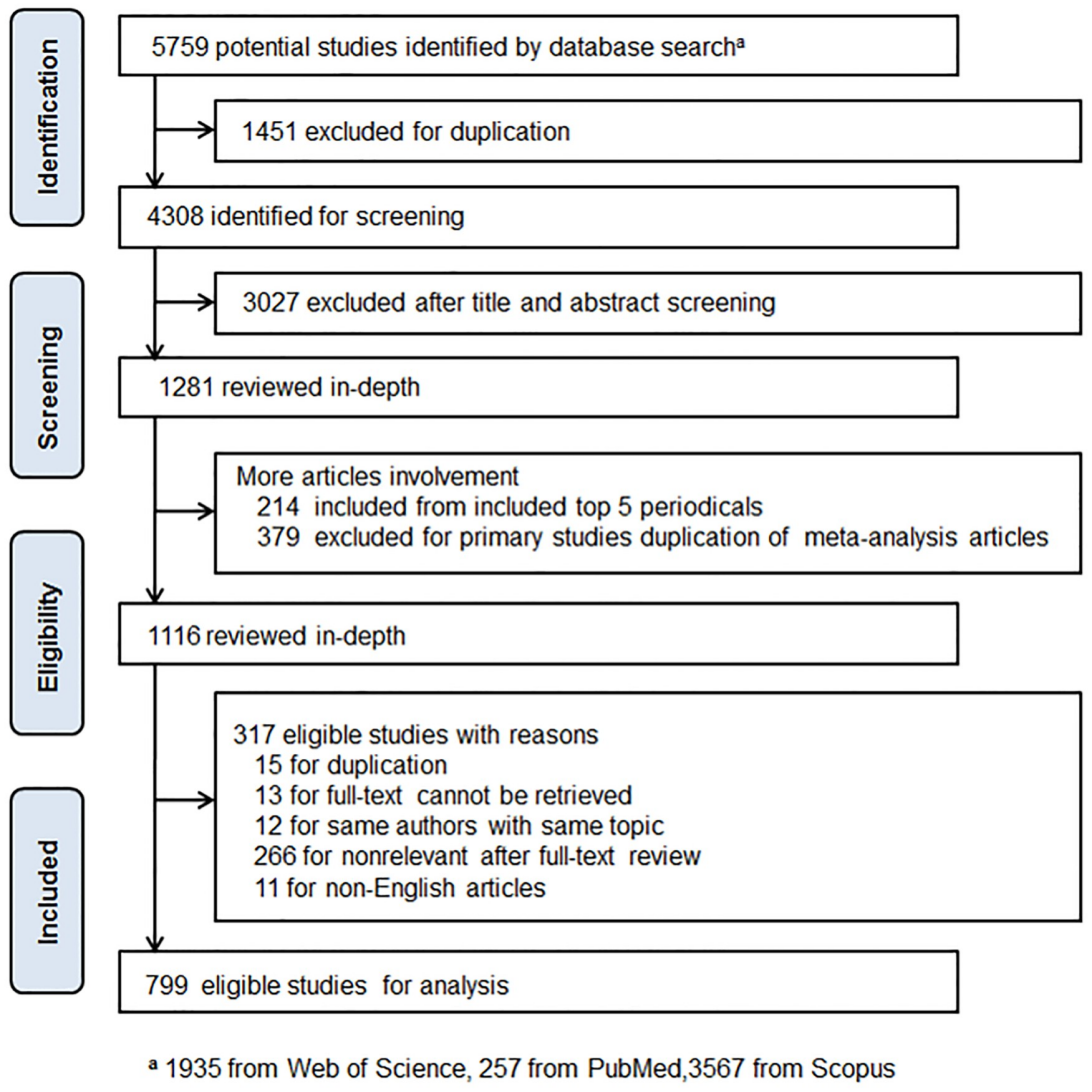
The diagram of eligible studies (inclusive articles) selection.

The original search was conducted in May of 2018; the Web of Science, PubMed, and Scopus databases were searched, resulting in a total of 5759 potentially relevant studies. After a de-duplication of 1451 studies and the application of the inclusion criteria, 3027 studies were assessed as being irrelevant and excluded based on readings of the titles and the abstracts. In the end, 1281 studies were assessed for in-depth full-text screening. To prevent overlooking potentially relevant papers, we manually screened the top five impact factor periodicals in the database we were searching. We traced the reference lists and the cited literatures of the included studies, and then we reviewed the newly collected literatures to generate more relevant studies. Further, after preliminary consultation with experts, we included studies on two additional health outcome categories, pregnancy and children and mental disorders. Hence, 214 more potential studies were included during this process. Besides, 379 original studies of the inclusive meta-analysis and systematic review studies were removed for duplication. In total, 1116 studies were included for in-depth full-text screening analysis and 799 eligible studies were included in the end. The detailed articles selection process was shown in Fig 1.

**Fig 2:**
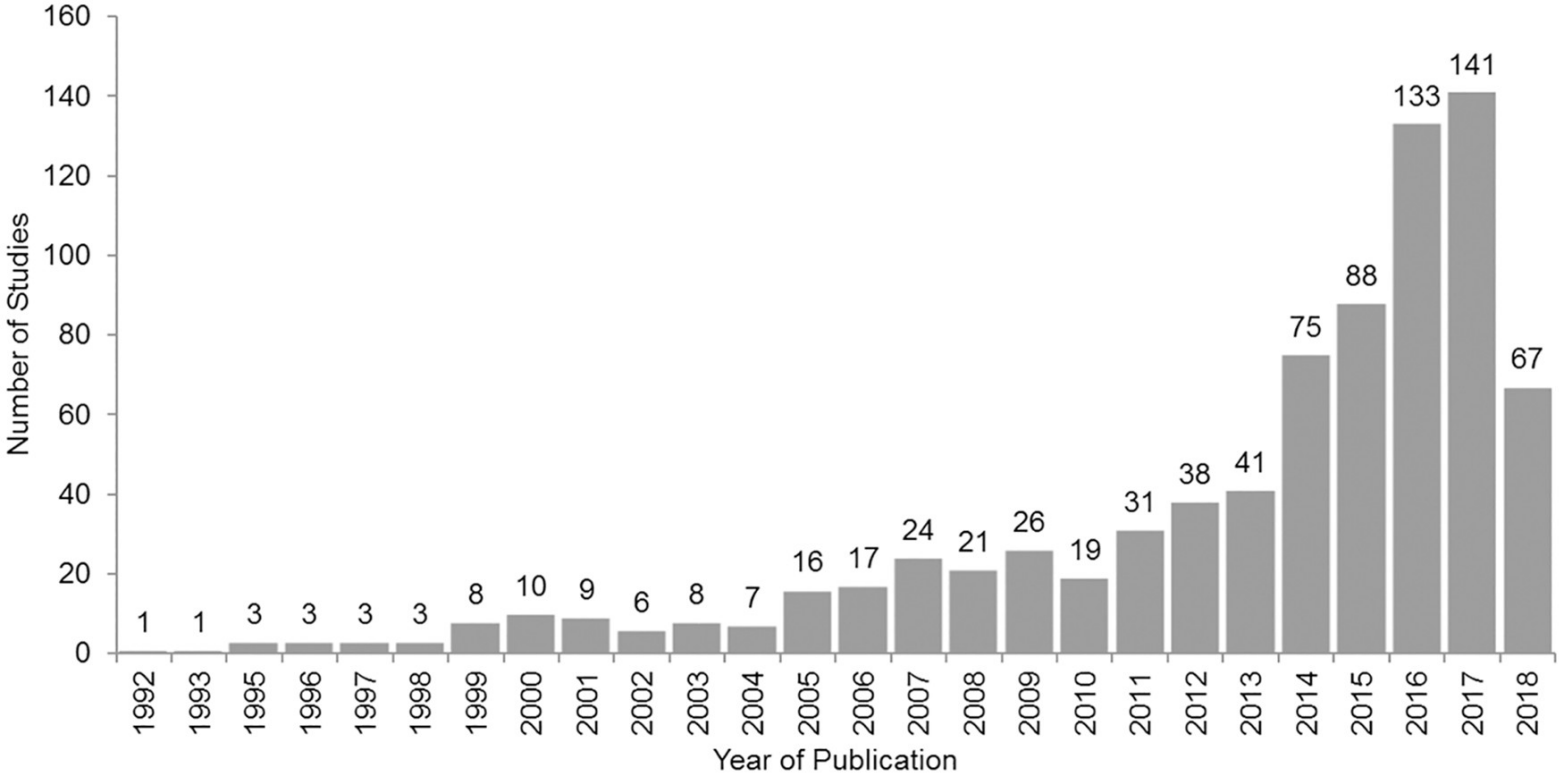
Number of inclusive studies published between 1992 and 2018. The included studies increased during this period, more than 75% of studies were published after 2011.

The included air pollution related health outcome studies increased between 1992 and 2018, as shown in Fig 2. Most studies were published in the last decade and more than 75% of studies (614/76.9%) were published after 2011.

The general characteristics summary of all included studies are shown in Table 3. Most studies were carried out in Asia, Europe and North America (280/35.0%, 261/32.7% and 219/27.4%, respectively). According to the category system of journal citation reports (JCR) in the Web of Science, 323/40.4% of all studies on health outcomes came from environmental science, 213/26.7% came from the field of medicine, and 24/3.0% were from economics. The top three research designs of the included studies were cohort studies, systematic reviews and meta-analyses, and time series studies (116/14.5%, 107/13.4% and 76/9.5%, respectively). Almost all included studies were published in journals (794/99.4%). The lengths of the included studies ranged from four pages [34] to over thirty-nine pages [35].

**Table 3:** General inclusive articles descriptions.

Study Characteristic (Total n = 799)		Count n (%)
**Year of publication**	1992–2000	32 (4.0)
	2001–2010	153 (19.2)
	2011–2015	273 (34.2)
	2016–2018	341 (42.7)
**Continent**	Asia	280 (35.0)
	Europe	261 (32.7)
	North America	219 (27.4)
	Australia or New Zealand	19 (2.4)
	South America	16 (2.0)
	Africa	4 (0.5)
**Discipline**	Environmental sciences	323 (40.4)
	Medicine	213 (26.7)
	Public environmental occupational health	139 (17.4)
	Environmental sciences & public environmental occupational health	52 (6.5)
	Multidisciplinary	30 (3.8)
	Economics	24 (3.0)
	Neurosciences	7 (0.9)
	Toxicology	6 (0.8)
	Pharmacology	2 (0.3)
	Biochemistry & molecular biology	1 (0.1)
	Geosciences	1 (0.1)
	Physiology	1 (0.1)
**Study design**	Cohort	116 (14.5)
	Meta-analysis and systematic reviews	107 (13.4)
	Time series	76 (9.5)
	Crossover	56 (7.0)
	Cross-sectional	31 (3.9)
	Qualitative (other/not specified)	413 (51.7)
**Type of publication**	Journal	794 (99.4)
	Conference paper	5 (0.6)

### Regions of studies

**Fig 3:**
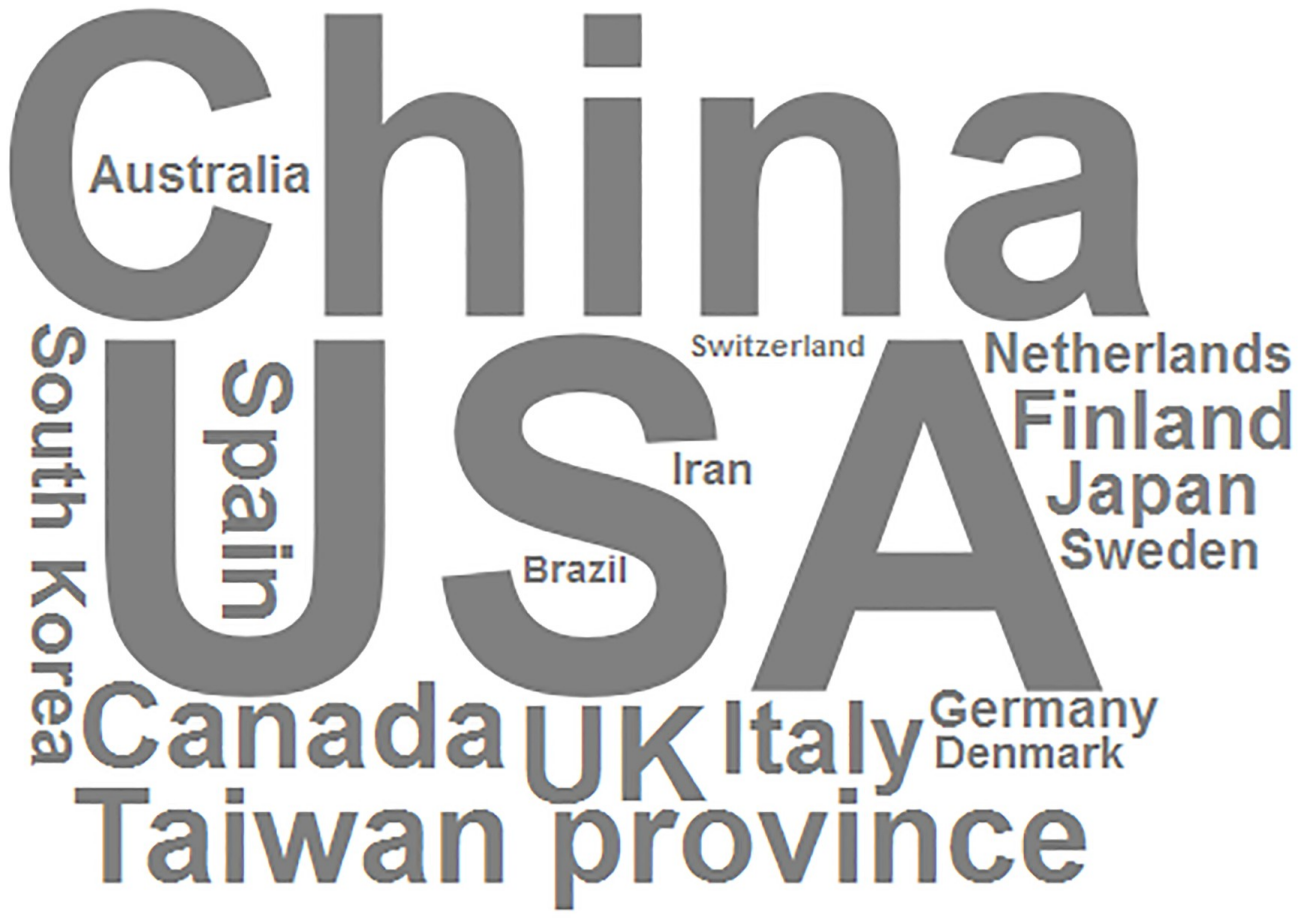
Word cloud of the country of included studies. Word cloud representing the country of included studies, the size of each term is in proportion to its frequency.

Table 4 outlines the locations in which health outcomes were affected by outdoor air pollution. The continents of Asia (277/34.7%), Europe (219/27.4%), and North America (168/21.0%) account for most of these studies. As the word cloud in Fig 3 illustrates, most of the included studies had been mainly conducted in the United States and China. About 62.8% of the studies (502) had been especially conducted in developed countries.

**Table 4:** Regions of the studies’ descriptions.

Region of Studies (Total n = 799)		Count n (%)
**Regions**	Asia	277 (34.7)
	Europe	219 (27.4)
	North America	168 (21.0)
	South America	20 (2.5)
	Australia or New Zealand	15 (1.9)
	Africa	4 (0.5)
	Not clearly mentioned	96 (12.0)
**Same as corresponding author’s address or not**	Same	573 (71.7)
	Different	127 (15.9)
	Mixed	64 (8.0)
	Not clearly mentioned	35 (4.4)
**Region country attribution**^a^	Developed country	502 (62.8)
	Developing country	196 (24.5)
	Developed & developing country	64 (8.0)
	Poor country	2 (0.3)
	Not clearly mentioned	35 (4.4)

^a^ The classification of countries was based on the version of the international monetary fund (IMF) list.

Most authors (573/799) evaluated the air pollution health outcomes of their own continent, at a proportion of 71.7%.

### Types of air pollution and related health outcomes

We categorized the health outcomes, by consulting with experts, into respiratory diseases, chronic diseases, cardiovascular diseases, health records, cancer, mental disorders, pregnancy and children, and other diseases (Table 5). We also divided the outdoor air pollution into general air pollution gas, fine particulate matter, other hazardous substances, and a mixture of them.

**Table 5:** Health outcome categories of outdoor air pollution.

Outcomes (Total n = 799)	Subgroups	General Air Pollution Gas	Fine Particulate Matter	Other Hazardous Substances	Mixed Air Pollution
**Respiratory diseases**	Asthma	14	9	4	42
	Allergic disease	-	2	-	7
	Lung diseases	7	11	-	20
	Respiratory symptoms	19	6	2	36
	COPD	1	3	-	8
	Wheeze	2	-	-	6
**Chronic diseases**	Diabetes	-	2	-	1
	Chronic disease	2	8	-	11
**Cardiovascular diseases**	Blood disease	3	5	-	14
	Cardiovascular disease	3	8	1	10
	Heart disease	4	8	-	11
	OHCA	-	-	-	2
	Stroke	3	5	-	12
	VTE	-	1	-	-
**Health records**	ER visits	9	6	-	20
	Hospital admissions	6	12	2	39
	Morbidity	3	1	-	15
	Mortality	24	69	3	67
	Outpatient visits	1	1	-	8
**Cancer**	Bladder Cancer	-	-	-	2
	Brain tumor	-	-	1	3
	Breast Cancer	-	-	1	5
	Liver cancer	-	-	-	1
	Lung cancer	1	8	1	11
	Cancer (not specified)	1	-	2	1
**Mental disorders**	Alzheimer’s disease	-	1	-	-
	Parkinson’s disease	1	1	-	4
	Depression & stress	-	-	-	4
	Annoyance	1	1	-	-
	ASD	-	-	1	1
	Cognitive function	1	4	-	7
	Mental (behavioral) disorder	1	-	-	-
**Pregnancy & Children**	Birth weight	4	2	-	6
	Infant death	1	-	-	1
	Infantile eczema	-	-	-	2
	Preterm Birth	2	2	-	5
	Fertility	-	-	-	2
	Pregnancy	3	4	-	3
	Pregnancy-induced hypertension	1	1	-	6
	Other diseases	3	1	-	3
**Other diseases**		10	25	2	45

COPD, chronic obstructive pulmonary disease; OHCA, out-of-hospital cardiac arrest; VTE, venous thrombo embolism; ER, emergency room; ASD, autism spectrum disorder.

**Fig 4:**
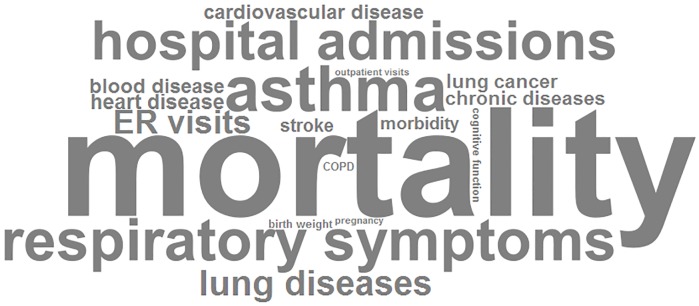
Word cloud of outdoor air pollution related health outcomes. Word cloud representing the health outcomes of included studies, the size of each term is in proportion to its frequency.

Most of the health records showed that mortality (163/286; 57.0%) was the most common health outcome related to outdoor air pollution, as is visually represented in Fig 4. Respiratory diseases (e.g., asthma and respiratory symptoms) and cardiovascular diseases (e.g., heart disease) that resulted from exposure to outdoor air pollution were also common (69/199, 63/199 and 23/90; or 34.7%, 31.7% and 25.6%; respectively).

### Types of affected groups

The population of included studies was categorized into seven subgroups: birth and infant, children, women and pregnancy, adults, elderly, all ages and not specified (Table 6). The largest air pollution proportion fell under the groups of all ages and children (261/799; 32.7% and 165/799; 20.7%), health outcomes of respiratory diseases in children account for the largest groups (114/199; 57.3%).

**Table 6:** Description of the types of affected groups.

Outcomes (Total n = 799)	Birth & Infant	Children	Women & Pregnancy	Adults	Elderly	All Ages	Not Specified
**Respiratory diseases**	4	114	1	23	11	33	13
**Chronic diseases**	-	2	7	3	4	8	-
**Cardiovascular diseases**	1	3	3	18	14	33	18
**Health records**	12	18	2	22	29	136	67
**Cancer**	-	-	9	1	2	20	6
**Mental disorders**	1	5	3	5	6	4	4
**Pregnancy & Children**	14	7	31	-	-	-	-
**Other diseases**	-	16	4	12	10	27	13

There were 121 research studies in the “Not Specified” group. As shown in Table 6, the “Birth & Infant,” “Women & Pregnancy,” “Children,” and “elderly” groups occupied the subject areas of more than half of the total included studies, which means that air pollution affected these population groups more acutely. Moreover, age is a confounding factor for the prevalence of cancer and cardiovascular diseases. However, there were only 2 studies (2/38, 5.3%) on cancer and 14 studies (14/90, 15.6%) on cardiovascular diseases in the elderly group.

### Summary of results

Of all included studies, 95.2% reported at least one statistically positive result, 4.4% were convincingly negative, and only 0.4% showed ambiguous results (Table 7).

**Table 7:** Health outcomes according to authors’ conclusions.

Conclusion	Total-n (%)	Respiratory Diseases	Chronic Diseases	Cardiovascular Diseases	Health Records	Cancer	Mental Disorders	Pregnancy & Children	Other Diseases
**Positive correlation**	761 (95.2)	191	22	82	277	37	25	48	79
**Not clear**	3 (0.4)	-	-	1	-	-	1	1	-
**No correlation**	35 (4.4)	8	2	7	9	1	2	3	3

There were 27 primary studies that showed no association between air pollution and disease, including cancer (n = 1), chronic diseases (n = 1), cardiovascular diseases (n = 6), health records (n = 7), pregnancy and children (n = 2), respiratory diseases (n = 6), mental disorders (n = 1), and other diseases (n = 3). Moreover, eight meta-analyses showed no evidence for any association between air pollution and disease prevalence (childhood asthma, chronic bronchitis, asthma, cardio-respiratory mortality, acute respiratory distress syndrome and acute lung injury, mental disorder, cardiovascular disease, and daily respiratory death). Three meta-analyses showed ambiguous results for mental health, venous thromboembolism, and hypertension.

## Discussion

Our scoping review provided an overview on the subject of outdoor air pollution and health outcomes. We adhered to the methodology outlined for publishing guidelines and used the six steps outlined by the scoping review protocol. The guiding principle ensured that our methods were transparent and free from potential bias. The strengths of the included studies are that they tend to focus on large sample sizes and broad geographical coverage. This research helped us to identify research gaps and disseminate research findings [23] to policymakers, practitioners, and consumers for further missing or potentially valuable investigations.

### Principal findings

Among the included studies, we identified various health outcomes of outdoor air pollution, including respiratory diseases, chronic diseases, cardiovascular diseases, health records, cancer, mental disorders, pregnancy and children, and other diseases. Among them, asthma in respiratory diseases and mortality in health records were the most common ones. The study designs contained cohort, meta-analysis, time series, crossover, cross-sectional, and other qualitative methods. In addition, we included economically relevant studies [12, 36, 37] to investigate the causal inference of outdoor air pollution on health outcomes. Further, pregnancy and children, mental disorders, and other diseases are health outcomes that might have uncertain or inconsistent effects. For example, Kirrane et al. [38] reported that PM_2.5_ had positive associations with Parkinson’s disease; however, some studies report that there is no statistically significant overall association between PM exposure and such diseases [39]. Overall, the majority of these studies suggested a potential positive association between outdoor air pollution and health outcomes, although several recent studies revealed no significant correlations [40–42].

### Time frame of included studies

The time frame of included studies is one of the most important characteristics of air pollution research. Even in the same country or region, industrialization and modernization caused by air pollution is distinguished between different time periods [43, 44]. In addition, the more the public understands environment science, the more people will take preventative measures to protect themselves. This is also influenced by time. Although air pollution should not be seen as an inevitable side effect of economic growth, time period should be considered in future studies. The publication trends with regard to air pollution related health outcome research increased sharply after 2010. In recent times, published studies have begun to pay more attention to controlling confounding factors such as socioeconomic factors and human behavior.

### Population and country

More than 50% of the studies on the relationship between air pollution and health outcomes originated from high income countries. There was less research (<25%) from developing countries and poor countries [45–48], which may result from inadequate environmental monitoring systems and public health surveillance systems. Less cohesive policies and inadequate scientific research may be another reason. In this regard, stratified analysis by regional income will be helpful for exploring the real estimates. It is reported that the stroke incidence is largely associated with low and middle income countries rather than with high income countries [49]. More studies are urgently needed in highly populated regions, such as Eastern Asia and North and Central Africa.

It is worth noting that rural and urban differences in air pollution research have been neglected. There are only eight studies focused on the difference of spatial variability of air pollution [50–57]. Variation is common even across relatively small areas due to geographical, topographical, and meteorological factors. For example, an increase in PM_2.5_ in Northern China was predominantly from abundant coal combustion used for heating in the winter months [58]. These differences should be considered with caution by urbanization and by region. Data analysis adjustment for spatial autocorrelation will provide a more accurate estimate of the differences in air. What’s more, in some countries such as China, migrants are not able to access healthcare within the cities; this has resulted in misleading conclusions about a “healthier” population and null based bias was introduced [59].

### Other studies (including systematic reviews and economic studies) on outdoor air pollution

Our scoping review included a large number of systematic reviews and meta-analyses. Of the included 107 systematic review and meta-analyses, the most discussed topics were respiratory diseases influenced by mixed outdoor air pollution [60–62]. Little systematic review research focused on chronic diseases, cancer, and mental disorders, which are current research gaps and potential research directions. A large overlap remains between the primary studies included in the systematic reviews. However, some systematic reviews that focused on the same topic have conflicting results, which were mainly caused by different inclusion criteria and subgroup analyses [63,64]. To solve this problem, it is critical that reporting of systematic reviews should retrieve all related published systematic reviews and meta-analyses.

As for the 24 included economic studies, two kinds of health outcomes—morbidity [65] and economic cost [66]—were discussed separately using regression approaches. The economic methods were different from those used in the epidemiology; the study focused on causal inference and provided a new perspective for examining the relevant environmental health problems. Furthermore, meta-regression methodology, an economic synthesis approach, proved to be very effective for evaluating the outcomes in a comprehensive way [67].

### Diagnostic criteria for diseases

The diagnostic criteria for diseases forms an important aspect of health-related outcomes. The diagnostic criteria for stroke and mental disorders might be less reliable than those for cancer, mobility, and cardiovascular diseases [68]. Few studies provided detailed disease diagnostic information on how the disease was measured. Thus, the overall effect estimation of outdoor air pollution might be overestimated. It is recommended that ICD-10 or ICD-11 classification should be adopted as the health outcome classification criterion to ensure consistency among studies in different disciplines considered in future research [69].

In spite of these broad disease definitions, studies in healthy people or individuals with chronic diseases were not conducted separately. People with chronic diseases were more susceptible to air pollution [70]. It is obvious that air pollution related population mobility might be underestimated. However, the obvious association of long-term exposure to air pollution with chronic disease related mortality has been reported by prospective cohort studies [71]. It should be translated to other diverse air pollution related effect research. The population with pre-existing diseases should be analyzed as subgroups.

Except for the overall population, subgroups of people with outdoor occupations and athletes [72,73], sensitive groups such as infants and children, older adults [74,75], and people with respiratory or cardiovascular diseases, should be analyzed separately.

### Measurement of personal exposure

The measurement of personal exposure to air pollutants (e.g., measurement of errors associated with the monitoring instruments, heterogeneity in the amount of time spent outdoors, and geographic variation) was lacking in terms of accurate determination. There is a need for clear reporting of these measurements. The key criterion to determine if there is causal relationship between air pollution and negative health outcomes was that at least one aspect of these could be measured in an unbiased manner.

### Pollutant dispersion factor

It is well known that the association between air pollution and stroke, and respiratory and cardiovascular disease subtype might be caused by many other factors such as temperature, humidity, season, barometric pressure, and even wind speed and rain [76–78]. These confounding factors related to aspects of energy, transportation, and socioeconomic status, may explain the varying effect size of the association between air pollution and diseases.

While the associations reported in epidemiological studies were significant, proving a causal relationship between the different air pollutants affected by any other factors and adverse effects has been more challenging. To avoid bias, these modifier effects should be compared with previous localized studies. In fact, how the confounding variables account for the heterogeneity should be explored by case-controlled study design or other causal interference research designs.

### Study limitations

The following limitations should not be overlooked. First, scoping reviews are based on a knowledge synthesis approach that allows for the mapping of gaps in the existing literature; however, they lack quality assessment for the included studies, which may be an obstacle for precise interpretation. Some improvements have been made by adding a quality assessment [15,22,79] to increase the reliability of the findings, and other included studies control for quality by including only peer-reviewed publications [80]; however, this is not a requirement for scoping reviews. While our paper aimed to comprehensively present a broader range of global-level current published literatures related to outdoor air pollution health outcomes, we did not assess the quality of the analyzed literature. The conclusions of this scoping review were based on the existence of the selected studies rather than their intrinsic qualities.

Second, bias is an inevitable problem from the perspectives of languages, disciplines, and literatures in knowledge synthesis. We included literatures from electronic databases, key journals, and reference lists to avoid “selection bias” and then included unpublished literature to avoid “publication bias”; further, we also conscientiously sampled among the studies to ensure that there was a safeguard against “researcher bias.” We only took English language articles into account because of the cost and time involved in translating the material, which might have led to a potential language bias [23]. However, in scoping reviews, language restriction does not have the importance that it does in meta-analysis [81].

## Conclusions

In all, the topic of outdoor air pollution exposure related health outcomes is discussed across multiple-disciplines. The various characteristics and contexts of different disciplines suggest different underlying mechanisms worth of the attention of researchers and policymakers. The presentation of the diversity of health outcomes and its relationship to outdoor exposure air pollution is the purpose of this scoping review for new findings in future investigations.

## Supporting information

S1 Table Literature search strategies. (PDF)

S2 Table PRISMA-ScR checklist. (PDF)
