# Supplementary material for: Exposure to outdoor air pollution and its human health outcomes: A scoping review
Source: PLoS One. 2019 May 16;14(5):e0216550. doi: 10.1371/journal.pone.0216550 (PMC6522200; doi:10.1371/journal.pone.0216550)
Supplement: S1 Table — (PDF) [file pone.0216550.s001.pdf]

**S1 Table. Literature search strategies.**

Search strategies for databases Web of Science.

| Database: Web of Science, Dates: Inception to May 22th, 2018 | | |
|---|---|---|
| # | Searches | Results |
| #1 | TS = (Ozone OR sulfur dioxide OR carbon monoxide OR nitrogen dioxide OR $PM_{2.5}$ OR $PM_{10}$ OR total suspended particle OR suspended particulate matter OR Toxic air pollutant OR volatile organic pollutant OR nitrogen oxide) | 669,588 |
| #2 | TS = (asthma OR lung cancer OR respiratory infection OR respiratory disorder OR diabetes OR chronic respiratory disease OR chronic obstructive pulmonary disease OR hypertension OR heart rate variability OR heart attack OR cardiopulmonary disease OR ischemic heart disease OR blood coagulation OR deep vein thrombosis OR stroke OR morbidity OR hospital admission OR outpatient visit OR emergency room visit OR mortality OR DNA methylation change OR neurobehavioral function OR Inflammatory disease OR skin disease OR abortion OR Alzheimer's disease OR disability OR cognitive function OR Parkinson's disease) | 7,128,708 |
| #3 | TS = (outdoor AND air pollution) | 10,703 |
| | #1 AND #2 AND #3 | 1,731 |
| #4 | TS = ("meta analysis ") | 173,028 |
| | #1 AND #2 AND #3 AND #4 | 53 |
| #5 | TS = ("systematic review ") | 143,227 |
| | #1 AND #2 AND #3 AND #5 | 198 |
| deduplication: 1892 | | |

Search strategies for databases PubMed.

| Database: PubMed, Dates: Inception to May 22th, 2018 | | |
|---|---|---|
| # | Searches | Results |
| #1 | Search ((((((((((Ozone[MeSH Terms]) OR sulfur dioxide[MeSH Terms]) OR carbon monoxide[MeSH Terms]) OR nitrogen dioxide[MeSH Terms]) OR $PM_{2.5}$[MeSH Terms]) OR $PM_{10}$[MeSH Terms]) OR total suspended particle[MeSH Terms]) OR suspended particulate matter[MeSH Terms]) OR Toxic air pollutant[MeSH Terms]) OR volatile organic pollutant[MeSH Terms]) OR nitrogen oxide[MeSH Terms] | 137,662 |
| #2 | Search (((((((((((((((((((((((((((asthma[MeSH Terms]) OR lung cancer[MeSH Terms]) OR respiratory infection[MeSH Terms]) OR respiratory disorder[MeSH Terms]) OR diabetes[MeSH Terms]) OR chronic respiratory disease[MeSH Terms]) OR chronic obstructive pulmonary disease[MeSH Terms]) OR hypertension[MeSH Terms]) OR | 3,111,918 |

| Database: PubMed, Dates: Inception to May 22th, 2018 | | |
|---|---|---|
| | heart rate variability[MeSH Terms]) OR heart attack[MeSH Terms]) OR cardiopulmonary disease[MeSH Terms]) OR ischemic heart disease[MeSH Terms]) OR blood coagulation[MeSH Terms]) OR deep vein thrombosis[MeSH Terms]) OR stroke[MeSH Terms]) OR morbidity[MeSH Terms]) OR hospital admission[MeSH Terms]) OR outpatient visit[MeSH Terms]) OR emergency room visit[MeSH Terms]) OR mortality[MeSH Terms]) OR DNA methylation change[MeSH Terms]) OR neurobehavioral function[MeSH Terms]) OR Inflammatory disease[MeSH Terms]) OR skin disease[MeSH Terms]) OR abortion[MeSH Terms]) OR Alzheimer’s disease[MeSH Terms]) OR disability[MeSH Terms]) OR cognitive function[MeSH Terms]) OR Parkinson's disease[MeSH Terms] | |
| #3 | Search (outdoor[Text Word]) AND air pollution[Text Word] | 3,132 |
| | #1 AND #2 AND #3 | 194 |
| #4 | meta analysis[Title/Abstract] | 109,835 |
| | #3 AND #4 | 44 |
| #5 | systematic review[Title/Abstract] | 120,881 |
| | #3 AND #5 | 39 |
| deduplication: 257 | | |

Search strategies for databases Scopus.

| Database: Scopus, Dates: Inception to May 30th, 2018 | | |
|---|---|---|
| # | Searches | Results |
| #1 | TITLE-ABS-KEY ( "Ozone" ) OR TITLE-ABS-KEY ( "sulfur dioxide" ) OR TITLE-ABS-KEY ( "carbon monoxide" ) OR TITLE-ABS-KEY ( "nitrogen dioxide" ) OR TITLE-ABS-KEY ( $PM_{2.5}$ ) OR TITLE-ABS-KEY ( $PM_{10}$ ) OR TITLE-ABS-KEY ( total AND suspended AND particle ) OR TITLE-ABS-KEY ( suspended AND particulate AND matter ) OR TITLE-ABS-KEY ( toxic AND air AND pollutant ) OR TITLE-ABS-KEY ( "volatile organic pollutant" ) OR TITLE-ABS-KEY ( "nitrogen oxide" ) | 324,714 |
| #2 | TITLE-ABS-KEY ( "asthma" ) OR TITLE-ABS-KEY ( "lung cancer" ) OR TITLE-ABS-KEY ( "respiratory infection" ) OR TITLE-ABS-KEY ( "respiratory disorder" ) OR TITLE-ABS-KEY ( "diabetes" ) OR TITLE-ABS-KEY ( "chronic obstructive pulmonary disease" ) OR TITLE-ABS-KEY ( "chronic obstructive pulmonary disease" ) OR TITLE-ABS-KEY ( "hypertension" ) OR TITLE-ABS-KEY ( "heart rate variability" ) OR TITLE-ABS-KEY ( "heart attack" ) OR TITLE-ABS-KEY ( "cardiopulmonary disease" ) OR TITLE-ABS-KEY ( "ischemic heart disease" ) OR TITLE-ABS-KEY ( "blood coagulation" ) OR TITLE-ABS-KEY ( "deep vein thrombosis" ) OR TITLE-ABS-KEY | 4,420,836 |

| Database: Scopus, Dates: Inception to May 30th, 2018 | | |
|---|---|---|
| | ( stroke ) OR TITLE-ABS-KEY ( morbidity ) OR TITLE-ABS-KEY ( " hospital admission" ) OR TITLE-ABS-KEY ( "outpatient visit" ) OR TITLE-ABS-KEY ( " emergency room visit" ) OR TITLE-ABS-KEY ( mortality ) OR TITLE-ABS-KEY ( " DNA Methylation Change" ) OR TITLE-ABS-KEY ( "neurobehavioral function" ) OR TITLE-ABS-KEY ( " Inflammatory disease " ) OR TITLE-ABS-KEY ( "skin disease" ) OR TITLE-ABS-KEY ( abortion ) OR TITLE-ABS-KEY ( "Alzheimer's disease" ) OR TITLE-ABS-KEY ( "skin disease" ) OR TITLE-ABS-KEY ( disability ) OR TITLE-ABS-KEY ( "cognitive function" ) OR TITLE-ABS-KEY ( "Parkinson's disease" ) | |
| #3 | ALL ( outdoor ) AND ALL ( " air pollution" ) | 30,841 |
| | #1 AND #2 AND #3 | 3,156 |
| #4 | TITLE-ABS-KEY ( "meta analysis" ) OR TITLE-ABS-KEY ( "systematic review" ) | 326,969 |
| | #1 AND #2 AND #3 AND #4 | 184 |
| deduplication: 3169 | | |

Search strategies for economic discipline.

| Database: Web of Science, Dates: Inception to May 30th, 2018 | | |
|---|---|---|
| # | Searches | Results |
| #1 | TS = (air +health) AND SO = (econom) | 43 |
| deduplication: 43 | | |

| Database: Scopus, Dates: Inception to May 30th, 2018 | | |
|---|---|---|
| # | Searches | Results |
| #1 | TITLE-ABS-KEY ( air ) AND TITLE-ABS-KEY ( health ) AND SRCTITLE ( econom ) | 398 |
| deduplication: 398 | | |
